# Curcumin Alleviates Sepsis‐Associated Acute Kidney Injury Potentially by Inhibiting Ferroptosis Through the ACSL4/GPX4 Signaling Pathway

**DOI:** 10.1002/ddr.70181

**Published:** 2025-10-27

**Authors:** Lifeng Wang, Nannan Wu, Weidong Zhang, Die Huang, Yongning Li

**Affiliations:** ^1^ Department of Emergency Medicine The First Affiliated Hospital of Dalian Medical University Dalian Liaoning China

**Keywords:** ACSL4/GPX4 axis, acute kidney injury, curcumin, ferroptosis, renal tubular epithelial cell, sepsis

## Abstract

This study aimed to investigate the protective effect of curcumin against sepsis‐associated acute kidney injury (SA‐AKI) in vitro and in vivo, and to clarify the role of ferroptosis in this protection. Human renal tubular epithelial cell (RTEC) line HK‐2 was stimulated with lipopolysaccharide (LPS). Ferroptosis was further induced with erastin. In vivo, a rat SA‐AKI model was produced by cecal ligation and puncture (CLP) and allocated to the Sham, sepsis, and curcumin (Cur) + sepsis (Sep) groups. Glutathione (GSH), malondialdehyde (MDA), and pro‐inflammatory cytokines were quantified by ELISA. Western blot analysis was used to evaluate the ferroptosis‐related proteins, including long‐chain acyl‐coenzyme synthetases 4 (ACSL4) and glutathione peroxidase 4 (GPX4). Apoptosis of RTECs was assessed with TUNEL staining, and ultrastructural changes were examined by transmission electron microscopy (TEM). Compared with the Sepsis group, the Cur + Sep group showed significantly lower Paller damage scores, reduced Scr, BUN, TNF‐α, IL‐1β, IL‐6, MDA, Fe^2+^ levels, and ACSL4 protein expression (all *p* < 0.05). Conversely, GSH and GPX4 levels were significantly elevated in the Cur + Sep group (both *p* < 0.05). TUNEL staining revealed fewer apoptotic RTECs in the Cur + Sep group compared with the Sepsis group (*p* < 0.05). TEM demonstrated swollen mitochondria with condensed membranes and vanished cristae in the sepsis group, changes that were markedly alleviated by curcumin. In HK‐2 cells, erastin abolished curcumin's protective effect against LPS‐induced injury. Curcumin attenuates SA‐AKI, likely by suppressing inflammation and ferroptosis via the ACSL4/GPX4 signaling pathway.

## 1 Introduction

Sepsis‐associated acute kidney injury (SA‐AKI) is a frequent and life‐threatening complication of sepsis that markedly raises mortality and the subsequent risk of chronic kidney disease. The condition stems from acute damage to glomerular and tubular cells precipitated by systemic inflammation, circulating toxins, and hemodynamic instability. Among renal elements, renal tubular epithelial cells (RTECs) are especially vulnerable to early‐stage ischemia and hypoxia, leading to loss of cell polarity, disruption of intercellular adhesion, and injury driven by autophagy, apoptosis, and necrosis (Liu et al. 2020; Hsu et al. 2019). Although the primary pathological changes in SA‐AKI occur in proximal tubular epithelial cells, the exact mechanisms underlying their injury remain poorly understood. Timely and appropriate management is crucial for improving clinical outcomes in patients with SA‐AKI; however, no specific therapeutic agents are currently available to address this condition.

Curcumin, a natural polyphenolic compound, exhibits multiple biological activities, including anti‐inflammatory, antioxidant, and free radical‐scavenging properties, for which it has been extensively studied for its therapeutic potential in cardiovascular, infectious, and oncological diseases (Wang et al. 2021; Zhao et al. 2023). Additionally, curcumin has been shown to alleviate ischemia‐reperfusion injury and protect organ function. Despite these beneficial effects, its role in ferroptosis, a regulated form of cell death dependent on iron overload, remains less explored, particularly in the context of SA‐AKI (Zhou and Zhang 2023; Liu et al. 2023a). Notably, ferroptosis has emerged as a key contributor to the death of RTECs, and inhibiting ferroptosis has been shown to improve renal function (Jia et al. 2023; Chen et al. 2022).

We conducted this study using cell culture and animal models of SA‐AKI to determine the effect of curcumin on SA‐AKI and to explore whether curcumin exerts its effects through the regulation of ferroptosis. The findings from this study may offer a theoretical foundation for the clinical management of SA‐AKI.

## 2 Materials and Methods

### 2.1 Reagents and Antibodies

Enzyme‐linked immunosorbent assay (ELISA) kits for interleukin‐1β (IL‐1β), interleukin‐6 (IL‐6), and tumor necrosis factor‐α (TNF‐α) were obtained from Wuhan Huamei Biotechnology Co. Ltd. (Wuhan, China). Curcumin and iron assay kits were purchased from Sigma‐Aldrich (USA). Kits for glutathione (GSH), malondialdehyde (MDA), blood urea nitrogen (BUN), and serum creatinine (Scr) were supplied by Nanjing Jiancheng Bioengineering Institute (Nanjing, China). The terminal deoxynucleotidyl transferase dUTP nick‐end labelling (TUNEL) apoptosis detection kit was acquired from Roche (Switzerland). The Cell Counting Kit‐8 (CCK‐8) and the DCFH‐DA reactive oxygen species (ROS) detection kit were sourced from Beyotime Biotechnology (Shanghai, China). Erastin was purchased from MedChemExpress (USA). Rabbit antibodies against GPX4, long‐chain acyl‐CoA synthetase‐4 (ACSL4), and GAPDH were obtained from Abcam (Cambridge, UK). Horseradish peroxidase (HRP)‐conjugated secondary antibodies were purchased from Zhongshan Golden Bridge Biotechnology Co. Ltd. (Beijing, China). The BCA assay kit and RIPA lysis buffer were obtained from Wuhan SanYing Biotechnology Co. Ltd. (Wuhan, China).

### 2.2 Cell Culture and Experimental Groups

THK‐2 cell line, a tubular epithelial cell line derived from normal human kidney tissue, was obtained from the Cell Bank of Peking Union Medical College Hospital (Beijing, China). HK‐2 cells were grown in a culture medium containing Dulbecco's Modified Eagle Medium (DMEM) supplemented with 10% fetal bovine serum and 1% penicillin‐streptomycin. The cultures were maintained at 37°C in a 5% CO₂ atmosphere with controlled humidity.

To assess the protective effects of curcumin on sepsis‐induced injury and the potential mechanism involving ferroptosis, HK‐2 cells were divided into four groups: (1) Control group; (2) Lipopolysaccharide (LPS) group; (3) Curcumin + LPS (Cur + LPS) group; and (4) Erastin + Curcumin + LPS (Erastin + Cur + LPS) group. HK‐2 cells in the LPS group were treated with LPS (2 μg/mL) for 24 h, as reported previously (Guo et al. 2022a). In the Cur + LPS group, after 24 h of treatment with LPS, curcumin at concentrations of 50, 100, and 200 μM was added and incubated for an additional 5 h to determine the optimal concentration. The dosage range was selected based on previous studies that reported protective effects of curcumin in various inflammatory and kidney injury models at doses between 50 and 200 mg/kg (Kar et al. 2020; Vieira et al. 2023). Following this, cells in the Erastin + Cur + LPS group were treated with 10 μM erastin, a ferroptosis inducer, according to the manufacturer's instructions and previously published studies utilizing 10 μM erastin to robustly initiate ferroptotic cell death in various renal and epithelial cell types. In this study, erastin‐induced ferroptotic stress enabled the evaluation of the protective effects of curcumin under such conditions for an additional 8 h. All experiments were carried out in triplicate.

Cell viability was assessed using the CCK‐8 assay according to the manufacturer's protocol. Briefly, HK‐2 cells were seeded in 96‐well plates at a density of 5 × 10³ cells/well and allowed to adhere overnight. After treatment with LPS, curcumin, and/or erastin for 24 h, 10 µL of CCK‐8 reagent was added to each well, and the plates were incubated at 37°C for 2 h in the dark. The optical density (OD) was measured at 450 nm using a microplate reader. Cell viability was calculated as a percentage relative to the control group.

### 2.3 Examination of Intracellular ROS Levels

HK‐2 cells were incubated with the fluorescent probe DCFH‐DA in serum‐free DMEM at 37°C for 30 min. After incubation, the cells were gently washed with phosphate‐buffered saline (PBS). Intracellular ROS levels were analyzed under an inverted fluorescence microscope, with green fluorescence intensity reflecting production. Quantification was carried out using ImageJ software (National Institutes of Health, USA).

### 2.4 Examination of Apoptosis in RTECs by Tunel Assay

The TUNEL assay was used to detect apoptotic cells. In brief, paraffin sections were deparaffinized in xylene and then rehydrated through a graded series of ethanol. After rinsing with PBS, each section was incubated with 25 μL of TUNEL reaction mixture at 37°C for 60 min, washed with PBS, and then incubated with 25 μL alkaline‐phosphatase‐conjugated antibody at 37°C for 30 min. Following a second PBS wash, one to two drops of BCIP/NBT chromogenic substrate were applied for 30 min. Sections were washed, air‐dried, counterstained with hematoxylin, sealed, and dried at 60°C. Apoptotic nuclei appeared brown or brown‐yellow under light microscopy. Eight random fields per slide were examined, and the apoptosis index was calculated as the percentage of apoptotic cells among total cells.

### 2.5 Animal Model of Sepsis‐Induced Acute Kidney Injury and Experimental Groups

All procedures involving handling of animals conformed to institutional and national guidelines for the care and use of laboratory animals. The study protocol for all animal experiments was reviewed and approved by the Animal Ethics Committee of Dalian Medical University (Approval No. AEE22098, approved on September 3, 2023).

Twenty‐four specific‐pathogen‐free male Sprague‐Dawley rats (6–8 weeks old, 200–220 g) were obtained from the Animal Experiment Center of Dalian Medical University (animal‐use license: SYXK (Liao) 2018‐0007; production license: SCXK (Liao) 2020‐0001). To evaluate curcumin's in vivo effect on sepsis‐induced injury, sepsis‐associated acute kidney injury (AKI) was induced by cecal ligation and puncture (CLP). Rats were randomly allocated to three groups (*n* = 8 per group): (1) Sham, (2) Sepsis, and (3) Curcumin + Sepsis (Cur + Sep; 200 mg/kg curcumin). Animals in the Sepsis and Cur + Sep groups were anesthetized with 1% pentobarbital sodium (60 mg/kg, intraperitoneally). After a 1.5 cm midline laparotomy, the cecum was exteriorized, ligated with 4–0 silk at its base, and punctured three times with a 20 G needle. A small amount of fecal material was gently extruded to ensure bacterial leakage before the cecum was returned to the abdomen. The abdominal wall was then closed in layers. Postprocedure, rats received a subcutaneous injection of physiological saline (10 mL/kg) for fluid resuscitation. Sham animals underwent the same procedure without cecal ligation or puncture. In the Cur + Sep group, curcumin (200 mg/kg) was injected intraperitoneally 1 h after CLP.

Postoperatively, all rats were kept individually with ad libitum access to food and water. At 24 h post‐surgery, the experimental rats were euthanized, and kidney tissues were collected for subsequent studies.

### 2.6 Determination of Cellular Iron Content

After treatment, HK‐2 cells from the control, LPS, Cur + LPS, and Erastin + Cur + LPS groups were harvested, centrifuged, and washed with PBS. Cell pellets were lysed in ice‐cold lysis buffer for 1 h, and the lysates were clarified by centrifugation at 13,000 rpm for 10 min. The supernatant was transferred to a 96‐well plate. Hydrochloric acid (0.1 mol/L) was added to each well, and the mixtures were incubated at 25°C for 30 min, followed by the addition of 100 µL iron‐detection reagent for an additional 60 min. Intracellular ferrous iron (Fe²⁺) was quantified at 562 nm with a microplate reader.

### 2.7 Histopathological Analysis of Renal Tissue

The left kidneys were fixed in 4% paraformaldehyde for 24 h and then dehydrated through a graded ethanol series. Tissues were embedded in paraffin and sectioned at 4 μm. After dewaxing and rehydration, the sections were stained with hematoxylin and eosin (H&E), mounted, and examined under a light microscope. Renal tubular injury was assessed via the Paller scoring method. At 200× magnification, ten lesion‐containing fields were randomly selected (100 tubules total). Scoring criteria were as follows: marked tubular dilation (1 point), brush‐border loss (1 point), cast formation (2 points), epithelial detachment (2 points), and exfoliated or necrotic cells within the lumen (1 point). The maximum score per tubule was 5.

### 2.8 Determination of MDA, GSH, and Fe2+ in the Kidney Tissue

The left kidney tissues were flash‐frozen in liquid nitrogen, ground into a powder, and weighed. Normal saline was added at a ratio of 1:9 by weight and volume. The mixture was centrifuged at 4°C and 3000 rpm for 15 min, and the supernatant was collected for analysis. According to the kit instructions, add the sample and the working liquid to be examined, mix them evenly, and then centrifuge. Detect the absorbance value of each sample, draw a standard curve, and calculate the concentrations of GSH, MDA, and Fe2+ .

### 2.9 Determination of TNF‐α, IL‐6, and IL‐1β Levels in Renal Tissue

The left kidney tissue was homogenized in a small volume of liquid nitrogen. After centrifugation at 3000 rpm, the supernatant was collected. Levels of the inflammatory cytokines IL‐1β, IL‐6, and TNF‐α were quantified using ELISA kits on a microplate reader according to the manufacturer's instructions and calculated from standard curves.

### 2.10 Transmission Electron Microscopy (TEM) of Renal Tissue Ultrastructure

Renal tissue was fixed in pre‐chilled 3% glutaraldehyde at 4°C for 12 h, rinsed three times in PBS (15 min each), post‐fixed in 1% osmium tetroxide for 2 h, and washed again in PBS. Specimens were dehydrated through a graded ethanol series and embedded in epoxy resin. Ultrathin sections were cut, placed on copper grids, and double‐stained with 4% uranyl acetate followed by 0.4% lead citrate. Ultrastructural analysis was performed with a Hitachi TEM (Hitachi High‐Technologies, Tokyo, Japan).

### 2.11 Western Blot Analysis of GPX4 and ACSL4 Protein Expression in Renal Tissue

Total protein was extracted from the renal tissues, and protein concentrations were quantified using a bicinchoninic acid (BCA) assay. Renal tissue samples were lysed using RIPA lysis buffer containing protease and phosphatase inhibitors. The tissues were homogenized on ice and lysed for 30 min, followed by centrifugation at 12,000 × g for 15 min at 4°C. The resulting supernatants were collected as total protein lysates. Protein concentrations were determined using a BCA protein assay according to the manufacturer's instructions. For Western blot analysis, 30 μg of total protein per sample was separated by 10% SDS‐PAGE and subsequently transferred to polyvinylidene difluoride (PVDF) membranes. The membranes were blocked with 5% nonfat milk for 1 h at room temperature (RT), followed by three washes with Tris‐buffered saline containing 0.1% Tween‐20 (TBST; 10 min per wash). The membranes were then incubated overnight at 4°C with primary antibodies at the following dilutions: GPX4 (1:2000), ACSL4 (1:2000), and GAPDH (1:2000). After washes with TBST, the membranes underwent incubation with horseradish peroxidase (HRP)‐conjugated secondary antibodies for 2 h at RT. Enhanced chemiluminescence (ECL) was used to visualize protein bands, and the band intensities were quantified using ImageJ software.

### 2.12 Statistical Analysis

All statistical analyses were performed using SPSS version 27.0 (IBM Corp., Armonk, NY, USA). Normally distributed data were presented as mean ± standard deviation (SD). One‐way analysis of variance (ANOVA) was used for multiple group comparisons, following a test to verify the homogeneity of variance. Post hoc analyses were conducted using the least significant difference *t*‐test for data with equal variances. Dunnett's T3 test was applied to data with unequal variances. A *p*‐value < 0.05 was considered statistically significant.

## 3 Results

### 3.1 Effect of Curcumin on HK‐2 Cell Viability

**Figure 1:**
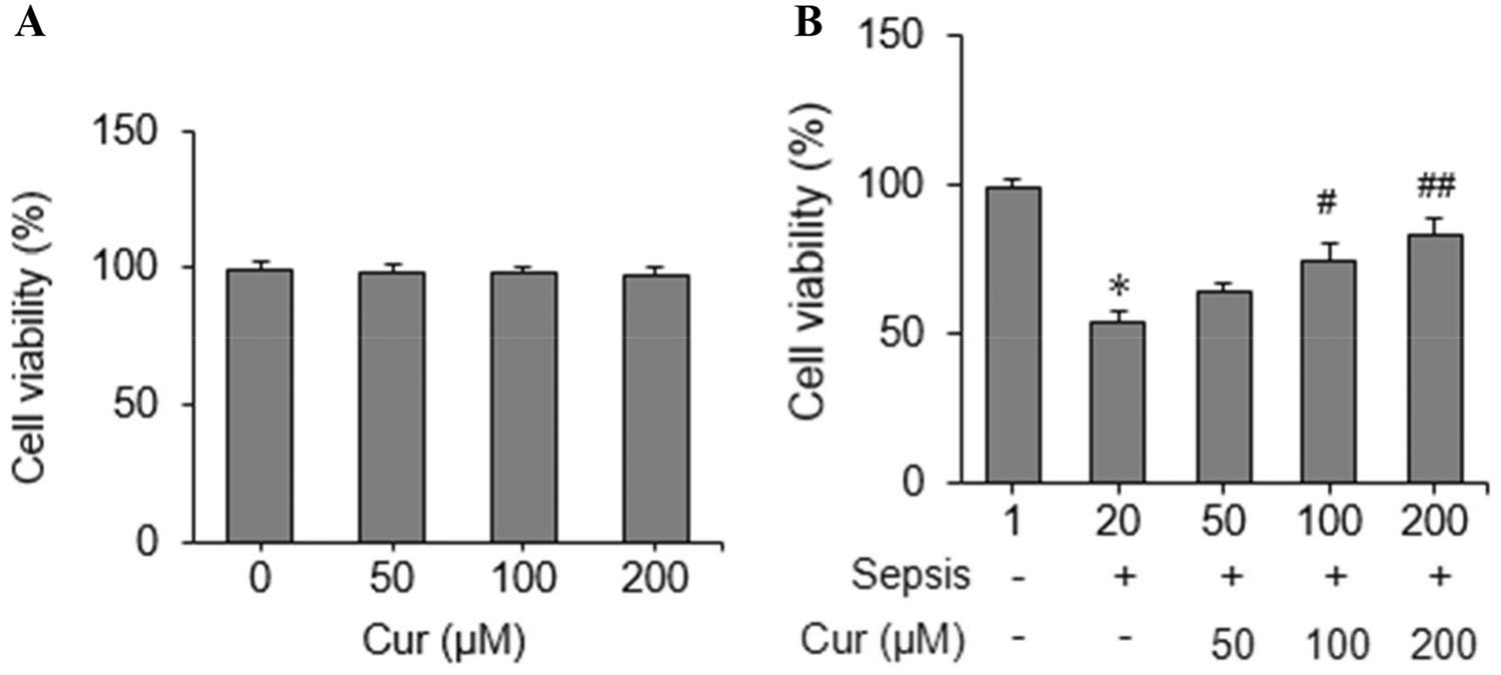
Effects of curcumin on the viability of HK‐2 cells. HK‐2 cells were treated with various concentrations of curcumin (Cur; 0, 50, 100, and 200 μM) with or without lipopolysaccharide (LPS) induction. Cell viability was assessed using the CCK‐8 assay. (A) Effects of curcumin on cell viability under normal conditions; (B) Effects of curcumin treatment on cell viability in LPS‐induced sepsis. Cells were pretreated with (+) or without (−) LPS, followed by curcumin treatment for an additional 5 h. *p < 0.05 versus control group; #*p* < 0.05 versus Sep + 0 μM Cur group; ##*p* < 0.05 versus Sep + 50 μM Cur group.

The effect of curcumin on HK‐2 cell viability was examined using the Cell Counting Kit‐8 (CCK‐8) assay. Curcumin at concentrations of 50, 100, and 200 μM showed no significant cytotoxicity to HK‐2 cells (Figure 1A). Curcumin treatment increased cell viability in HK‐2 cells pretreated with LPS in a dose‐dependent manner (Figure 1B). Based on these results, the concentration of 200 μM was selected for performing subsequent experiments.

### 3.2 Effects of Curcumin on ROS and Fe²⁺ Levels in HK‐2 Cells

**Figure 2:**
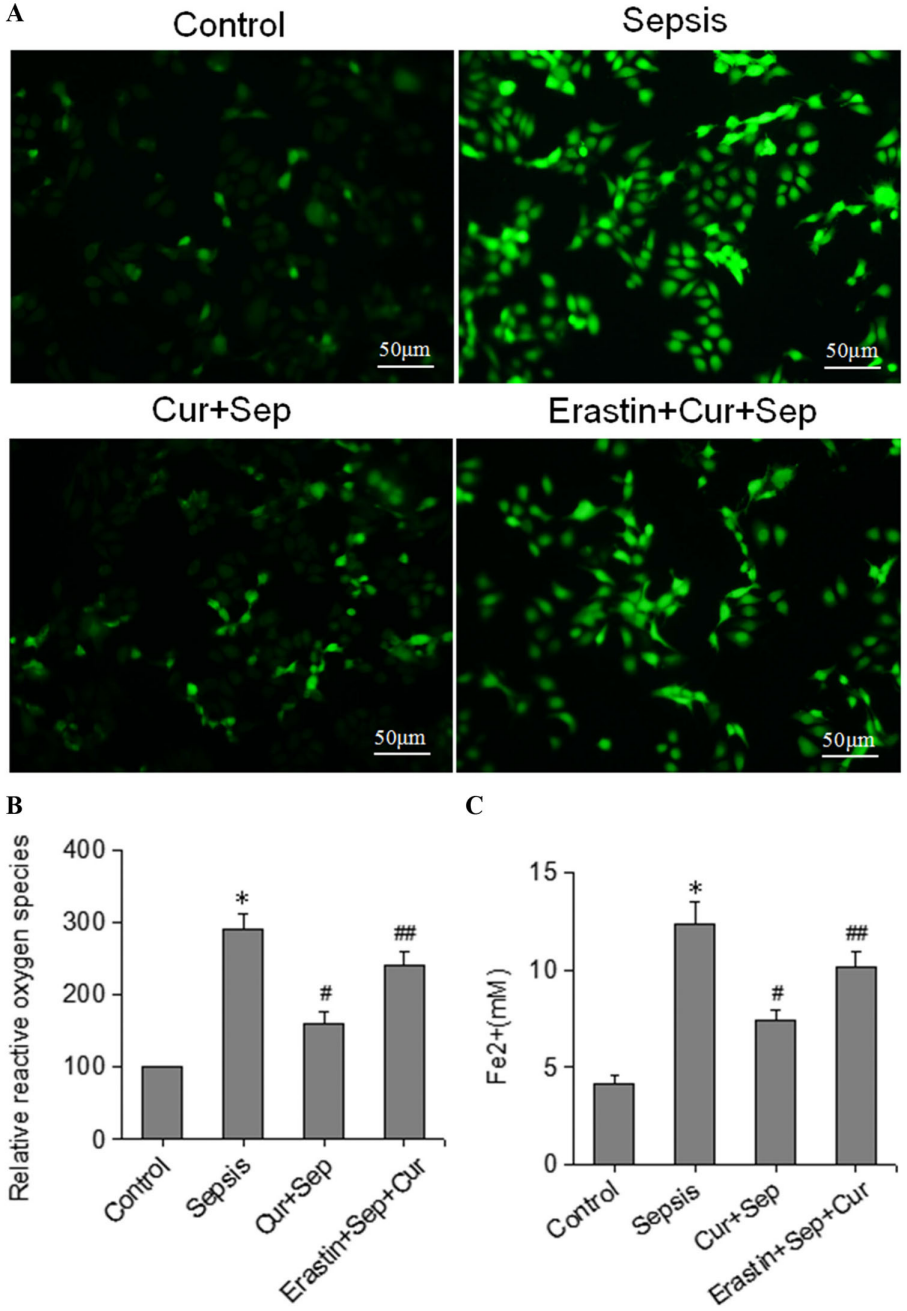
Intracellular ROS and Fe²⁺ levels in HK‐2 cells across experimental groups. The four groups included: Control (untreated cells), Sepsis (LPS treatment), Cur + Sep (LPS pretreatment for 24 h), followed by 200 μM curcumin for 5 h); and Erastin + Cur + LPS (LPS pretreatment for 24 h), then 200 μM curcumin for 5 h, followed by 10 μM erastin for 8 h. (A) Intracellular ROS levels measured by DCFH‐DA staining; (B) Intracellular Fe²⁺ levels. *p < 0.05 compared to the Control group; #*p* < 0.05 compared to the Sepsis group; ##*p* < 0.05 compared to the Cur + Sep group.

As shown in Figure 2A,B, ROS levels in HK‐2 cells were significantly elevated following LPS treatment in the Sepsis group versus the Control group. Curcumin treatment in the Cur + Sep group markedly attenuated this LPS‐induced increase in ROS compared with the Sepsis group (*p* < 0.05). Furthermore, the induction of ferroptosis with its inducer, Erastin, in the Erastin + Cur + Sep group significantly elevated ROS levels compared to the Cur + Sep group (*p* < 0.05).

Intracellular Fe²⁺ levels were measured in the four experimental groups. As illustrated in Figure 2C, Fe²⁺ levels in the Sepsis group were significantly greater than those in the Cur + Sep group (*p* < 0.05). Similarly, Fe²⁺ levels in the Erastin + Cur + Sep group were sufficiently higher than in the Cur + Sep group (*p* < 0.05), yet significantly lower in comparison with the Sepsis group (*p* < 0.05).

These findings indicate that erastin augments ROS production and promotes ferroptosis in HK‐2 cells. Including the Erastin + Cur + Sep group, therefore, provided a stringent test of curcumin's efficacy under heightened ferroptotic stress; the data confirm curcumin's protective capacity even when ferroptosis was actively triggered.

### 3.3 Effects of Curcumin on Histopathological Features and Renal Function in Rats

Light microscopy revealed the normal findings of renal tissues in the Sham group, characterized by intact glomeruli and RTECs. In contrast, the sepsis group showed pathological alterations, including interstitial edema, RTECs swelling with indistinct boundaries, inflammatory cell infiltration, intertubular hyperemia, tubular necrosis, and cast formation.

**Figure 3:**
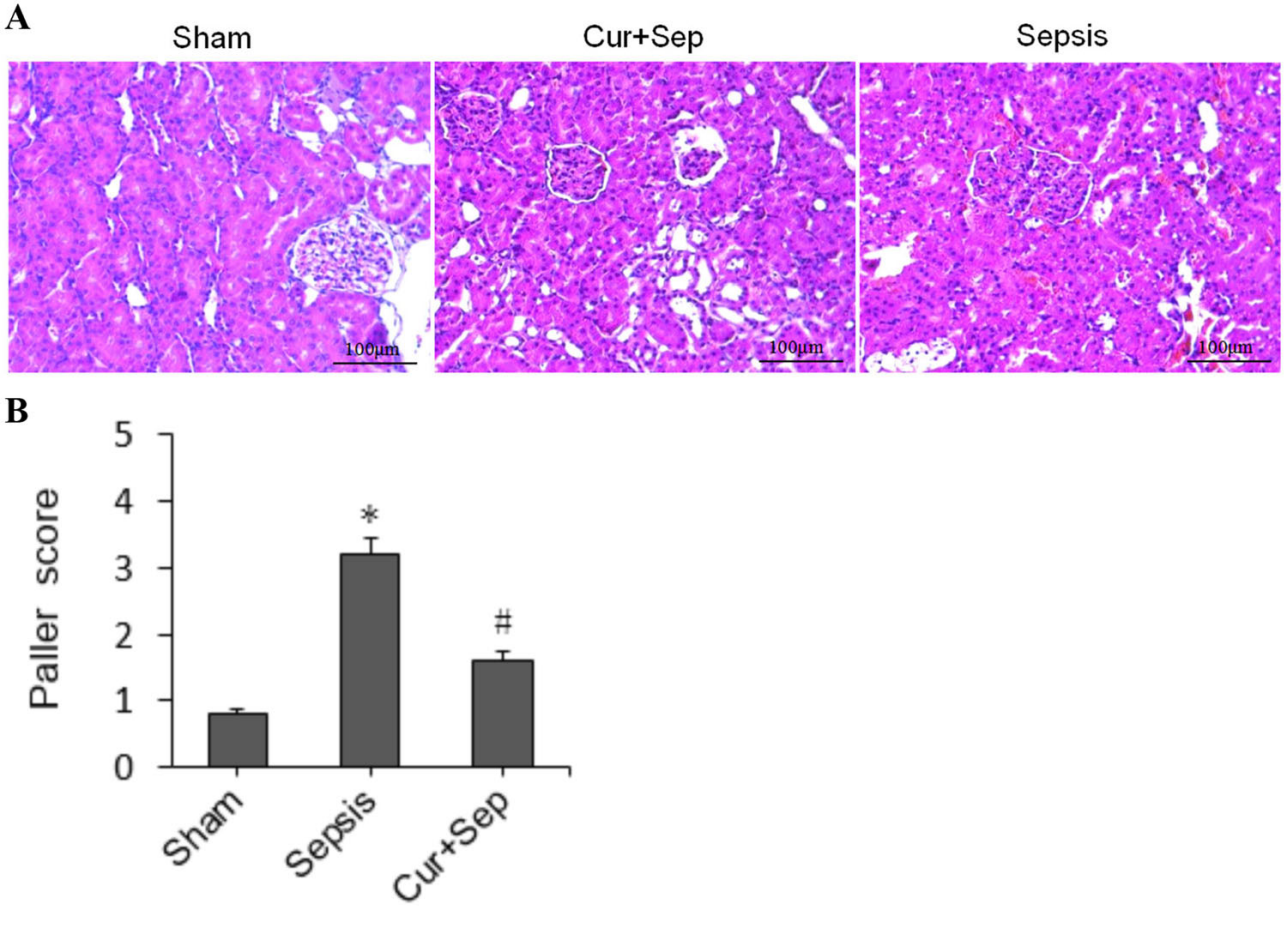
Histopathological analysis of renal tissues in experimental rats. (A) Representative histopathological images; (B) Quantitative analysis of Paller scores. Experimental groups included: Sham (sham‐operated control), Sepsis [cecal ligation and puncture (CLP)], and Cur + Sep (CLP with 200 mg/kg curcumin treatment). *p < 0.05 compared to the Sham group; #*p* < 0.05 compared to the Sepsis group.

Figure 3A,B and Table 1 show that the sepsis group had significantly higher serum Scr and BUN concentrations than the Sham group (both *p* < 0.05). Paller injury scores were likewise greater in the sepsis group (*p* < 0.05). Curcumin administration (Cur + Sep) markedly mitigated histopathological damage and reduced Scr, BUN, and Paller scores compared with sepsis (all *p* < 0.05). These findings demonstrate that curcumin mediates the attenuation of renal injury in vivo (Figure 3A,B; Table 1).

**Table 1:** Levels of BUN and Scr in three groups of rats.

Groups	BUN (μmol/L)	Scr (μmol/L)
Sham	8.58 ± 1.36	43.32 ± 3.45
Sepsis	36.76 ± 3.18*	138. 25 ± 8.52*
Cur + Sep	16.48 ± 2.64^#^	94.48 ± 6.88^#^

*Note:* Data were expressed as mean ± standard deviation, with *n* = 8 rats in each group. Compared with the Sham group, *p < 0.05; compared with the sepsis group, #*p* < 0.05.

### 3.4 Effects of Curcumin on Renal MDA, GSH, and Fe²⁺ Levels in Rats

The renal MDA, GSH, and Fe²⁺ levels in the Sham, sepsis, and Cur + Sep groups were compared, and the results are presented in Table 2. The sepsis group exhibited significantly higher MDA and Fe²⁺ levels, along with significantly lower GSH levels (all *p* < 0.05). In comparison, curcumin treatment resulted in a significant reduction in MDA and Fe²⁺ levels, as well as an increase in GSH levels, in the Cur + Sep group compared to the sepsis group (all *p* < 0.05).

**Table 2:** Renal MDA, GSH, and Fe2+ levels in three groups of rats.

Groups	MDA (nmol/mg)	GSH (nmol/g)	Fe2+ (mg/g)
Sham	4.23 ± 0.56	12.4 ± 1.18	0.42 ± 0.07
Sepsis	14.64 ± 1.36*	4.55 ± 0.76*	0.86 ± 0.13*
Cur + Sep	9.68 ± 1.18#	8.87 ± 0.93#	0.69 ± 0.11#

*Note:* Data were presented as mean ± standard deviation, with *n* = 8 rats in each group. Compared with the Sham group, *p < 0.05; compared with the sepsis group, #*p* < 0.05.

### 3.5 Effects of Curcumin on Renal TNF‐α, IL‐6, and IL‐1β in Rats

Renal concentrations of TNF‐α, IL‐6, and IL‐1β were assessed in the Sham, sepsis and Cur + Sep groups (Table 3). The sepsis group exhibited significantly higher levels of all three cytokines compared to the Sham group (*p* < 0.05). Curcumin treatment (Cur + Sep) markedly reduced each cytokine compared with the Sepsis group (*p* < 0.05).

**Table 3:** Comparison of TNF‐α, IL‐6, and IL‐1β levels in the kidney tissues of rats in each group.

Groups	TNF‐α (pg/ug)	IL‐6 (pg/ug)	IL‐1β (pg/ug)
Sham	4.74 ± 1.14	1.68 ± 0.26	9.52 ± 1.12
Sepsis	14.63 ± 2.18*	6.52 ± 0.76*	68.26 ± 5.34*
Cur + Sep	8.82 ± 1.85#	3.73 ± 0.47#	42.45 ± 3.85#

*Note:* Data were presented as mean ± standard deviation, with *n* = 8 rats in each group. Compared with the Sham group, *p < 0.05; compared with the sepsis group, #*p* < 0.05.

### 3.6 Effects of Curcumin on RTEC Apoptosis in Rats

**Figure 4:**
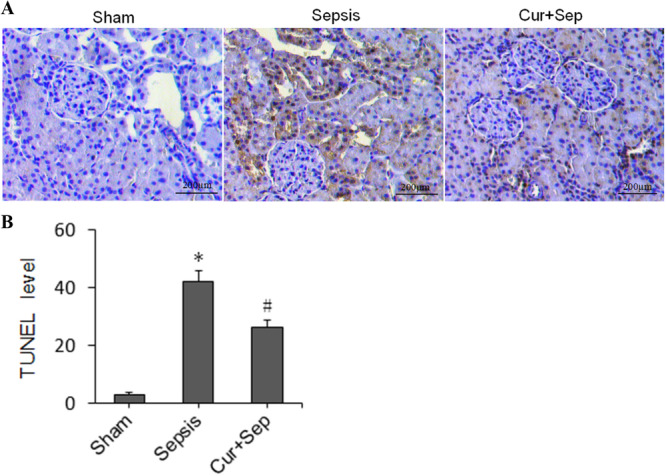
Apoptosis analysis of renal tubular epithelial cells in experimental rats. (A) Representative TUNEL assay images; (B) Quantitative analysis of apoptotic cells (apoptosis index) in three groups, including Sham (sham‐operated control), Sepsis [cecal ligation and puncture (CLP)], and Cur + Sep (CLP with 200 mg/kg curcumin treatment). *p < 0.05 compared to the Sham group; #*p* < 0.05 compared to the Sepsis group.

RTECs in the Sham group appeared light blue with intact and clear morphology. In contrast, the sepsis group exhibited a large number of apoptotic RTECs, characterized by brown or brown‐yellow granules, with an apoptosis index of 42.42% ± 3.47%. In the Cur + Sep group, the apoptotic cell number was significantly decreased in comparison with the sepsis group, with an apoptosis index of 26.43% ± 2.63%, which was significantly lower than that of the sepsis group but significantly higher than that of the Sham group (3.12% ± 0.68%) (all *p* < 0.05) (Figure 4A,B).

### 3.7 Comparison of Key Ferroptosis‐Related Proteins (ACSL4 and GPX4) in Rat Renal Tissues

**Figure 5:**
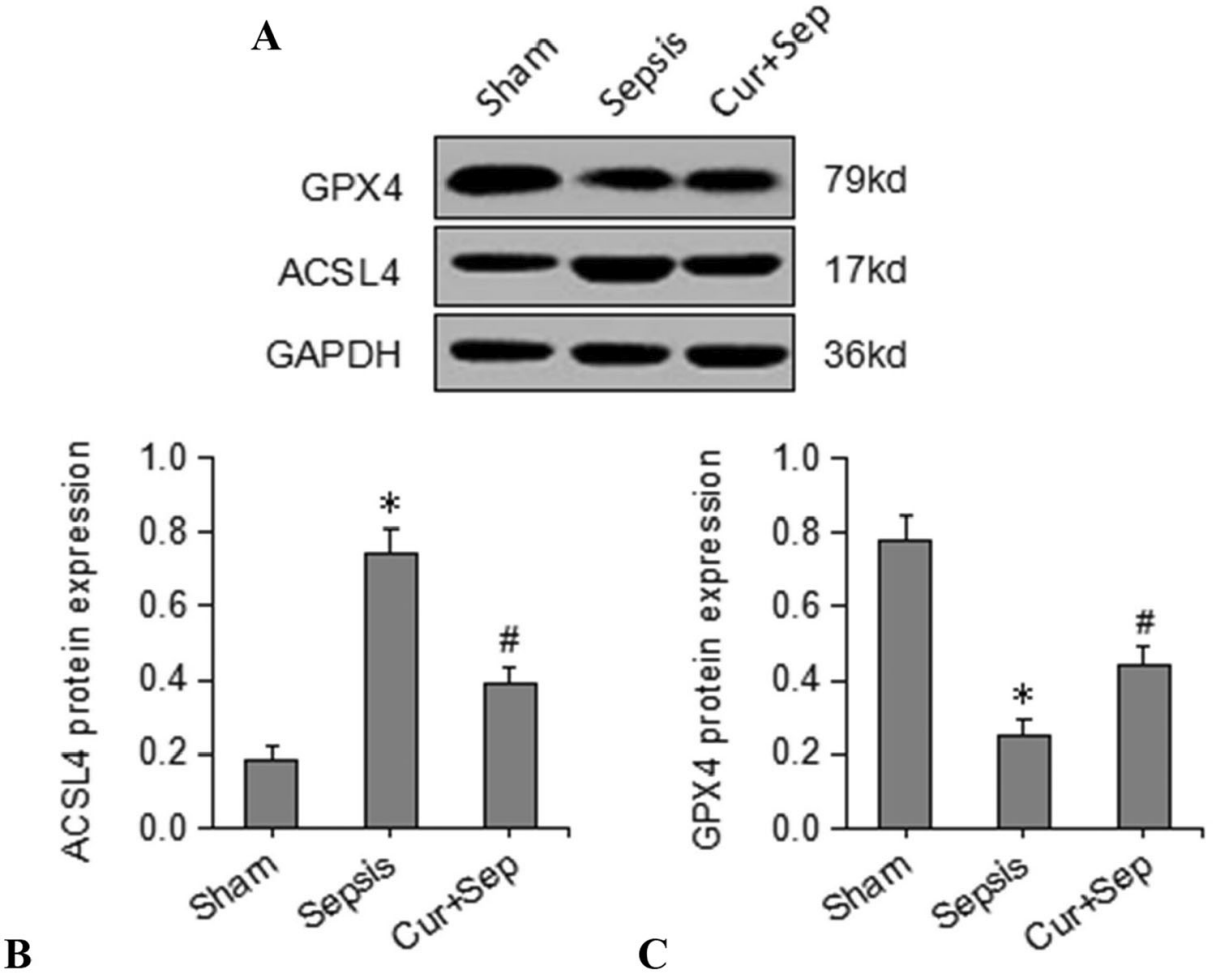
Renal ACSL4 and GPX4 protein expression levels in rats. (A) Representative Western blot images of ACSL4 and GPX4 protein levels; (B) Quantitative analysis of protein expression levels in three experimental groups: Sham (sham‐operated control), Sepsis [CLP], and Cur + Sep (CLP with 200 mg/kg curcumin treatment). *p < 0.05 compared to the Sham group; #*p* < 0.05 compared to the Sepsis group.

Western blot analysis was conducted to examine the expression of key ferroptosis‐related proteins, including ACSL4 and GPX4, and the results are demonstrated in Figure 5A–C. The expression level of ACSL4 in the Cur + Sep group was 38.6% ±  4.5%, significantly higher than in the Sham group (18.3% ±  3.4%) and significantly lower than in the Sepsis group (74.6% ±  6.5%) (*p* < 0.05) (Figure 5A,B). Additionally, the expression level of GPX4 in the Cur + Sep group was 44.2% ± 5.3%, significantly lower than that in the Sham group (78.2% ± 6.3%) and significantly greater compared to that in the Sepsis group (24.6% ± 4.5%) (*p* < 0.05; Figure 5A–C).

### 3.8 Ultrastructural Changes in Renal Tissues of Rats

**Figure 6:**
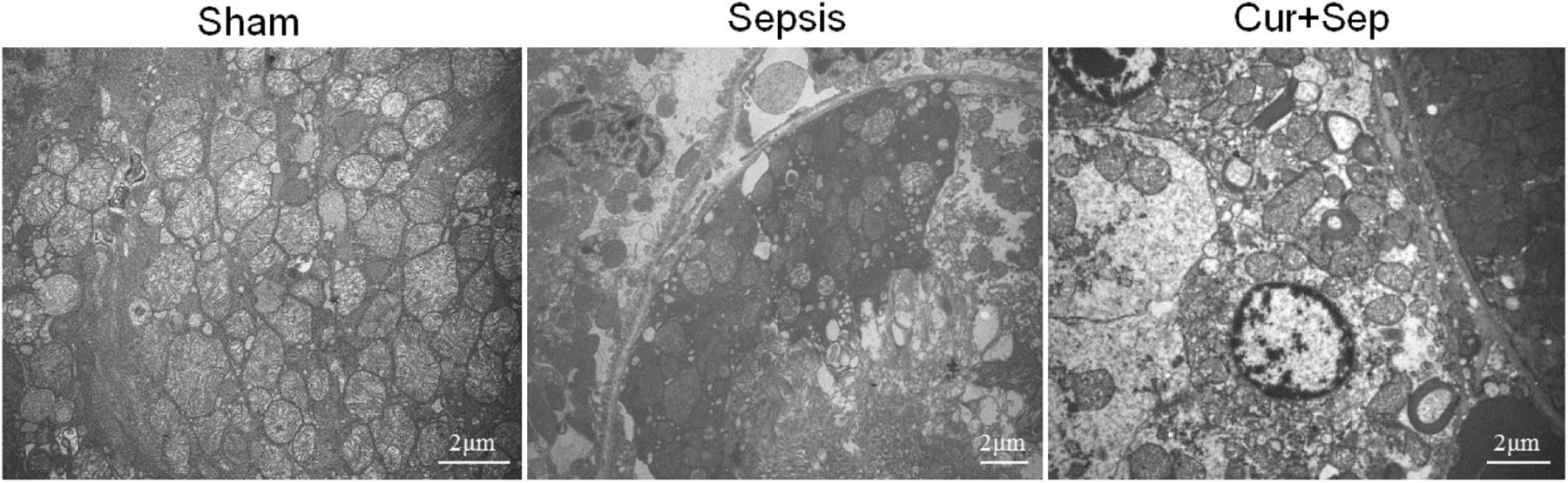
Ultrastructural analysis of rat renal tissues by transmission electron microscopy. Experimental groups: Sham (sham‐operated control), Sepsis (sepsis via CLP), and Cur + Sep (CLP with 200 mg/kg curcumin treatment).

TEM revealed intact structures in the RTECs of the Sham group, characterized by uniformly distributed nuclear chromatin and clearly defined mitochondria with complete contours. In contrast, the sepsis group exhibited mitochondrial damage, including shrinkage of mitochondria, increased membrane density, disruption of cristae, as well as partial vacuolization, with normal nuclear morphology. In the Cur + Sep group, some mitochondria displayed mild swelling and reduced or partially disrupted cristae; however, the overall morphology of the RTECs remained relatively preserved (Figure 6).

## 4 Discussion

SA‐AKI represents a potentially life‐threatening complication of sepsis, frequently observed in critically ill patients. Timely therapeutic intervention is crucial to mitigate renal dysfunction and improve prognosis (Privratsky et al. 2023; Zheng et al. 2023), yet effective treatments are still limited. In this study, utilizing in vitro and in vivo models of SA‐AKI, we demonstrated that curcumin exerted protective effects by attenuating injury, at least in part through the suppression of ferroptosis. Our findings suggested the therapeutic potential of curcumin in SA‐AKI.

In this study, analysis using DCFH‐DA fluorescent probe on HK‐2 cells revealed excessive production of ROS in the sepsis group, which was markedly reduced following curcumin treatment. However, ROS levels increased again after the addition of the ferroptosis inducer erastin. This suggests that intracellular iron overload results in excessive ROS production, which exerts toxic and damaging effects on cellular membranes. As the mitochondrial double‐membrane structure is a key site for ferroptosis, excessive ROS disrupts both plasma and organelle membranes, ultimately leading to the initiation of ferroptosis. Erastin, a classical ferroptosis inducer, was used in this study to simulate a condition of enhanced ferroptotic stress.

Several studies have reported that SA‐AKI is strongly associated with the release of inflammatory cytokines, oxidative stress, and apoptosis (Zhang et al. 2023a; Guo et al. 2022b; Zhang and Peng 2022). In our rat model of SA‐AKI, curcumin significantly lowered the pro‐inflammatory cytokines IL‐1β, TNF‐α, and IL‐6, as well as serum Scr and BUN. H&E staining revealed that the sepsis group exhibited pronounced pathological alterations, including interstitial edema, inflammatory cell infiltration, local congestion, and swelling of RTECs. The TUNEL assay indicated increased apoptosis in RTECs, and ETM demonstrated mitochondrial swelling, cristae rupture, and partial vacuolization, suggesting that mitochondria are primary targets of ROS‐induced injury. In contrast, the Cur + Sep group exhibited markedly attenuated renal damage, with a significantly lower Paller score than the sepsis group. Collectively, these findings indicate that curcumin confers renoprotective effects by mitigating inflammation, suppressing oxidative stress, and preserving mitochondrial function, particularly in the context of ferroptosis‐associated renal injury during sepsis.

Ferroptosis, a newly recognized form of regulated cell death dependent on iron overload, is mechanistically distinct from apoptosis, necrosis, and autophagy. Its primary feature is iron‐dependent lipid peroxidation (LPO), which leads to the accumulation of intracellular MDA and ultimately triggers ferroptotic cell death (Cui et al. 2021). MDA is a byproduct of LPO resulting from oxidative metabolism and serves as an indirect indicator of intracellular ROS levels. GSH is a critical intracellular antioxidant and ROS scavenger, as well as a key regulator of ferroptosis (Bayır et al. 2023). GSH acts as a substrate for GPX4, an enzyme that protects cells from LPO. By maintaining GPX4 activity, GSH effectively suppresses ferroptosis. Conversely, excessive depletion of GSH leads to GPX4 inactivation, resulting in increased LPO and subsequent ferroptosis. In the present study, curcumin treatment significantly reduced MDA but increased GSH in the kidney tissues of septic rats, suggesting that curcumin inhibits ferroptosis by attenuating LPO and scavenging ROS. Similarly, a recent study reported that a ceria nanozyme–curcumin co‐delivery system (CeCH) exerted potent antioxidative, anti‐ferroptotic, and anti‐inflammatory effects in a mouse model of sepsis‐induced cardiac injury, further supporting the therapeutic value of curcumin in multiorgan protection during sepsis (Jiang et al. 2024). Moreover, similar antioxidant and anti‐ferroptotic effects of curcumin have been reported by Liu et al. demonstrating that curcumin alleviates ferroptosis in experimental AKI in mice by inhibiting monoamine oxidase A (MAOA), thereby supporting its therapeutic potential against kidney injury (Liu et al. 2024).

ACSL4 is a key enzyme in fatty‐acid metabolism. It catalyzes the incorporation of long‐chain polyunsaturated fatty acids into membrane phospholipids, thereby promoting LPO and serving as a major driver of ferroptosis. Downregulation of ACSL4 inhibits ferroptosis, whereas its overexpression is associated with enhanced production of pro‐inflammatory cytokines (IL‐6, TNF‐α, and IL‐1β), and elevated lipid‐peroxide levels (Zhang et al. 2021).

In the present study, ACSL4 expression was markedly upregulated in SA‐AKI, while curcumin treatment significantly downregulated ACSL4 and concomitantly reduced inflammatory cytokine concentrations and LPO markers. These findings indicate that curcumin suppresses ferroptosis in SA‐AKI by limiting ACSL4‐mediated LPO. It has also been demonstrated that curcumin attenuates ferroptosis and oxidative stress in other ischemia/reperfusion‐related organ injuries. For instance, in a rat model of IR‐induced AKI, curcumin and the lipoxygenase inhibitor LoxBlock‐1 were found to mitigate ferroptotic damage in the heart, liver, and pancreas through regulating the ACSL4/GPX4 pathway (Kar et al. 2023). Although our study did not assess the effects of curcumin on distant organs, these findings establish its ferroptosis‐inhibiting effects and support its systemic therapeutic potential in critical conditions like SA‐AKI. The regulatory role of ACSL4 in ferroptosis and cytokine production has also been corroborated in recent investigations (Xiao et al. 2024). In addition, GPX4, a membrane‐associated enzyme responsible for lipid repair, serves as a crucial negative regulator of ferroptosis and plays an essential role in controlling oxidative damage during septic AKI (Liu et al. 2023b). Under septic conditions, large amounts of inflammatory cytokines (e.g., IL‐6) are released. IL‐6 not only contributes to renal oxidative stress but also inhibits GPX4 synthesis (Zhang et al. 2023b). Clinically, a recent prospective study indicated that ferroptosis‐related ACSL4 and GPX4 proteins are significantly dysregulated in septic ICU patients. Elevated ACSL4 levels were positively correlated to disease severity scores (SOFA and APACHE II) and 28‐day mortality, whereas GPX4 levels were inversely related to adverse outcomes. These findings provide robust clinical evidence in support of the role of ferroptosis in sepsis pathophysiology and highlight the potential of GPX4 and ACSL4 as prognostic biomarkers in SA‐AKI (Zeng et al. 2025). GPX4 utilizes GSH to neutralize lipid peroxides; however, GSH depletion results in GPX4 inactivation, leading to the accumulation of lipid peroxides. Furthermore, iron overload exacerbates ferroptosis. Intracellular Fe²⁺ accumulation contributes to the formation of an unstable iron pool, which, through the Fenton reaction, promotes ROS generation and LPO, ultimately resulting in ferroptosis (Zhou et al. 2023). Thus, iron accumulation in renal tissue is a critical pathophysiological feature of ferroptosis in septic RTECs. Our results showed that GPX4 protein expression in the Cur + Sep group was significantly higher than that in the sepsis group, while renal Fe²⁺ and MDA levels were significantly lower. These findings indicate that curcumin alleviates ferroptosis by upregulating GPX4 activity, enhancing GSH levels, and reducing iron overload and LPO, thereby exerting a protective effect on the kidney.

This study has several limitations. First, the Erastin + sepsis group was excluded, which limited the experimental design. Nonetheless, comparative analyses among the sepsis, Cur + sepsis, and Erastin + Cur + sepsis groups provide indirect evidence for the protective effect of curcumin against ferroptosis‐induced injury. Second, although experiments in cell culture and animal models of SA‐AKI indicate that curcumin attenuates renal injury by inhibiting ferroptosis via the GPX4 and ACSL4 pathways, direct confirmation through the specific inhibition or overexpression of GPX4 and ACSL4 is still required.

## 5 Conclusion

Curcumin exerts renoprotective effects in cell culture and rat models of SA‐AKI, most likely by inhibiting ferroptosis through the ACSL4/GPX4 pathway. These findings support the therapeutic potential of curcumin for the clinical management of SA‐AKI.

## Conflicts of Interest

The authors declare no conflicts of interest.

## Data Availability

The data that support the findings of this study are available from the corresponding author upon reasonable request.
